# An Efficient Method for Computing Expected Value of Sample Information for Survival Data from an Ongoing Trial

**DOI:** 10.1177/0272989X211068019

**Published:** 2021-12-30

**Authors:** Mathyn Vervaart, Mark Strong, Karl P. Claxton, Nicky J. Welton, Torbjørn Wisløff, Eline Aas

**Affiliations:** Department of Health Management and Health Economics, University of Oslo, Oslo, Norway; Norwegian Medicines Agency, Oslo, Norway; School of Health and Related Research, University of Sheffield, Sheffield, UK; Centre for Health Economics, University of York, York, UK; Department of Economics and Related Studies, University of York, York, UK; Population Health Sciences, University of Bristol, Bristol, UK; Department of Community Medicine, UiT The Arctic University of Norway, Oslo, Norway; Norwegian Institute of Public Health, Oslo, Norway; Department of Health Management and Health Economics, University of Oslo, Oslo, Norway

**Keywords:** bayesian decision theory, computational methods, economic evaluation model, expected value of sample information, generalized additive model, model averaging, Monte Carlo methods, nonparametric regression, survival data

## Abstract

**Background:**

Decisions about new health technologies are increasingly being made while trials are still in an early stage, which may result in substantial uncertainty around key decision drivers such as estimates of life expectancy and time to disease progression. Additional data collection can reduce uncertainty, and its value can be quantified by computing the expected value of sample information (EVSI), which has typically been described in the context of designing a future trial. In this article, we develop new methods for computing the EVSI of extending an existing trial’s follow-up, first for an assumed survival model and then extending to capture uncertainty about the true survival model.

**Methods:**

We developed a nested Markov Chain Monte Carlo procedure and a nonparametric regression-based method. We compared the methods by computing single-model and model-averaged EVSI for collecting additional follow-up data in 2 synthetic case studies.

**Results:**

There was good agreement between the 2 methods. The regression-based method was fast and straightforward to implement, and scales easily to include any number of candidate survival models in the model uncertainty case. The nested Monte Carlo procedure, on the other hand, was extremely computationally demanding when we included model uncertainty.

**Conclusions:**

We present a straightforward regression-based method for computing the EVSI of extending an existing trial’s follow-up, both where a single known survival model is assumed and where we are uncertain about the true survival model. EVSI for ongoing trials can help decision makers determine whether early patient access to a new technology can be justified on the basis of the current evidence or whether more mature evidence is needed.

**Highlights:**

- Decisions about new health technologies are increasingly being made while trials are still in an early stage, which may result in substantial uncertainty around key decision drivers such as estimates of life-expectancy and time to disease progression. Additional data collection can reduce uncertainty, and its value can be quantified by computing the expected value of sample information (EVSI), which has typically been described in the context of designing a future trial.
- In this article, we have developed new methods for computing the EVSI of extending a trial’s follow-up, both where a single known survival model is assumed and where we are uncertain about the true survival model. We extend a previously described nonparametric regression-based method for computing EVSI, which we demonstrate in synthetic case studies is fast, straightforward to implement, and scales easily to include any number of candidate survival models in the EVSI calculations.
- The EVSI methods that we present in this article can quantify the need for collecting additional follow-up data before making an adoption decision given any decision-making context.

## Introduction

The expected value of sample information (EVSI) quantifies the expected value to the decision maker of reducing uncertainty through the collection of additional data,^1,2^ for example, a future randomized controlled trial. Although a few studies have considered the use of EVSI methods at interim analyses of adaptive trials,^3^ overall little research has been done on EVSI for trials that are ongoing at the point of decision making.

In the past decade, the European Medicines Agency has introduced regulatory mechanisms that are aimed at accelerating the licensing of new pharmaceuticals, such as adaptive pathways^4^ and conditional marketing authorizations.^5^ When evidence is obtained from a trial at an early stage, the events of interest, such as disease progression or death, may have only been observed in a small proportion of patients. Health care authorities therefore have to issue guidance on new pharmaceuticals based on less mature evidence than previously, resulting in greater uncertainty about clinical and cost-effectiveness. With this comes an increased risk of recommending a technology that reduces net health benefit.^6^

Additional evidence can be valuable as it can lead to better decisions that improve health and/or reduce resource use.^6^ Positive adoption decisions can be costly or difficult to reverse and may remove the incentives for manufacturers to provide additional data. When a trial is ongoing at the point of decision making, for example, when follow-up is continued for regulatory purposes, there may therefore be value in delaying the adoption decision until additional data have been collected in the ongoing trial and uncertainty has reduced.^7^ In this context, there will be a tradeoff between granting early access to a new technology that may turn out to reduce health benefits and waiting for uncertainty to be reduced through ongoing data collection with a potential loss of health benefits while waiting. When the manufacturer is already committed to continuing the ongoing trial, the option to delay a decision is relevant even in a policy context in which the decision maker does not have the formal authority to commission research. The value of delaying the decision could be quantified, at least in theory, by computing the EVSI for the additional follow-up data.

Estimates of life expectancy and time to disease progression are often key drivers of cost-effectiveness, particularly in oncology. However, immature data means that there may be substantial uncertainty around these estimates, and they rely on extrapolation beyond the trial follow-up period.^8^ The choice of the survival distribution for extrapolation can have major implications for cost-effectiveness, and uncertainty surrounding this choice can be accounted for by model averaging, which may improve the quality of the extrapolations compared with selecting a single model.^9^ A potential benefit of continuing an ongoing trial is to reduce the structural uncertainty as to the most appropriate survival distribution. However, to the best of the authors’ knowledge, there is no guidance on how to compute EVSI for survival data from a trial that is ongoing at the point of decision making nor on how to account for structural uncertainty about the choice of survival model in the EVSI calculations.

In this article, we present algorithms for computing the EVSI of extending a trial’s follow-up with and without accounting for structural uncertainty. The algorithms are based on nested Markov Chain Monte Carlo (MCMC) methods and a fast nonparametric regression-based method.^10^ The nonparametric regression-based method^10^ is generally more practical than other EVSI approximation methods, as it neither requires nested Monte Carlo computations nor importance sampling.^11^ The article is structured as follows. In the second section, we describe single-model and model-averaged EVSI algorithms for survival data from an ongoing trial. In the third section, we compare the EVSI algorithms in 2 illustrative case studies, and in a final section, we conclude with a brief discussion.

## Method

### EVSI for an Ongoing Study Collecting Time-to-Event Data

#### Decision problem and model definition

We assume a decision problem with d=1,…,D decision options. The net benefit of option d is NB(d,θ) , and we have a cost-effectiveness model that predicts this quantity, given a vector of p possibly correlated model input parameters, $\theta =\{\theta_{1},\ldots ,\theta_{p}\}$ . Our current judgments about the vector θ is represented by the joint probability distribution p(θ) . Our goal is to choose the decision option with the greatest net benefit.

#### EVSI for further follow-up in an ongoing study

The EVSI for a new study that will provide (as yet uncollected) data, x , is defined as:

(1) $$\mathrm{EVSI}(\mathrm{new}\;\mathrm{study})=E_{x}[\underset{d}{\max}E_{\theta |x}\{\mathrm{NB}(d,\theta )\}]-\underset{d}{\max}E_{\theta }\{\mathrm{NB}(d,\theta )\},$$

where the first term is the expected value of a decision based on our beliefs about θ given the new data, p(θ|x) , and the second term is the expected value of a decision based on our beliefs about θ given current information alone, p(θ) .^12^ We now imagine that data x have been collected during a given follow-up period for this study, which we denote as time $t_{1}$ . This could be an interim analysis or the end of the study follow-up period.

The value of extending the follow-up from current time $t_{1}$ to some future point $t_{2}$ is given by

(2) $$\mathrm{EVSI}(\mathrm{ongoing}\;\mathrm{study})=E_{\tilde{x}|x}[\underset{d}{\max}E_{\theta |x,\tilde{x}}\{\mathrm{NB}(d,\theta )\}]-\underset{d}{\max}E_{\theta |x}\{\mathrm{NB}(d,\theta )\},$$

where the first term is the expected value of a decision based on our beliefs about θ given both new data, $\tilde{x}$ , collected between $t_{1}$ and $t_{2}$ , and data, x , collected between time 0 and $t_{1}$ . The second term is the expected value of a decision based on our beliefs about θ given only the information collected up until $t_{1}$ . See Appendix A for a fuller explanation.

#### Specifying current beliefs about model parameters for an ongoing study

The distribution for the cost-effectiveness model parameters given knowledge at $t_{1}$ p(θ|x) can be defined either in a fully Bayesian manner, by updating (possibly vague) prior information about θ with data x , or by fitting a standard frequentist statistical model to x and obtaining the maximum likelihood estimate for θ along with some expression of uncertainty and treating this as a Bayesian posterior. In the absence of strong prior information about θ , the 2 methods will produce very similar distributions for p(θ|x) , even with relatively little data.^13^

#### Specifying the likelihood for ongoing time-to-event data and left truncation

To compute EVSI, we must define the data-generating distribution for the follow-up data between $t_{1}$ and $t_{2}$ , $p(\tilde{x}|\theta )$ . We first consider the structure of the data we will observe. We assume our study has 2 arms, new treatment and standard care, and that N participants are recruited into each arm. Data, x , collected from time 0 to $t_{1}$ , take the form of a vector of times to death, time to end of follow-up, or time to loss to follow-up, whichever is soonest. Survival times for those alive at $t_{1}$ are censored. If we continue to collect data $\tilde{x}$ from $t_{1}$ to $t_{2}$ , we may observe times to death for the participants whose observations were censored at $t_{1}$ . Survival times for those alive at $t_{2}$ or lost to follow-up are now the only observations censored. Table 1 illustrates the structure of the data for 1 arm of a study with follow-up at 12 and 24 mo.

**Table 1:** Structure of Data for 1 Arm of a Study with Follow-up at 12 and 24 mo^a^

ID	Follow-up at $t_{1}=12$ Mo			Follow-up at $t_{2}=24$ Mo		Outcome
	Survival Time	Censoring Indicator, δ	At Risk at $t_{2}$	Survival Time	Censoring Indicator, δ	
1	9.3	1	No	—	—	Died at 9.3 mo
2	12.0^b^	0	Yes	13.4	1	Died at 13.4 mo
3	12.0^b^	0	Yes	24.0^b^	0	Alive at 24.0 mo
4	6.7^b^	0	No	—	—	LFU at 6.7 mo
5	12.0^b^	0	Yes	15.9^b^	0	LFU at 15.9 mo

LFU, lost to follow-up.

a Five participants are shown. Data are denoted x={(9.3,12,12,6.7,12),(1,0,0,0,0)} for observations up until $t_{1}=12$ mo and $\tilde{x}=\{(13.4,24,15.9),(1,0,0)\}$ for observations between $t_{1}$ and $t_{2}=24$ mo.

b Observation censored (δ=0) .

Survival times are usually assumed to arise from a data-generating process that can be described using a parametric model, the form of which must be chosen by the analyst.^14^ Censoring is common when collecting time-to-event data, as the follow-up time may not be long enough to observe the endpoint of interest for all individuals in the trial, and some individuals may be lost to follow-up.^15^ The likelihood function for survival data, x , obtained up until $t_{1}$ for a model with hazard function h(·) and survivor function S(·) , is

(3) $$\mathrm{Likelihood}\;p(x|\theta )=\overset{n_{1}}{\underset{i=1}{\Pi }}h(x_{i},\theta {)}^{\delta_{i}}S(x_{i},\theta ),$$

where i indexes the $n_{1}=N$ study participants at risk at time 0, where the censoring indicator $\delta_{i}=1$ when $x_{i}$ is an observed event, $\delta_{i}=0$ when $x_{i}$ is a censored observation, and where θ are the parameters of the survival distribution. The observed data set at time point $t_{1}$ consists of the $n_{1}$ survival times and censoring indicators, $x=\{x_{1},\ldots ,x_{n_{1}},\delta_{1},\ldots ,\delta_{n_{1}}\}$ .

The data collected between time points $t_{1}$ and $t_{2}$ is denoted $\tilde{x}=\{{\tilde{x}}_{1},\ldots ,{\tilde{x}}_{n_{2}},{\tilde{\delta }}_{1},\ldots ,{\tilde{\delta }}_{n_{2}}\}$ , where $n_{2}$ is the number of study participants at risk at $t_{1}$ . The likelihood function for $\tilde{x}$ is left truncated at $t_{1}$ to reflect that events beyond $t_{1}$ are conditional on not having occurred prior to $t_{1}$ .^16^ Unlike censoring, which contributes to the likelihood by plugging in a survival factor for censored observations as well as observed survival times, truncation does not add any data points to the likelihood. This distinction is important, because we want to avoid double counting the observed data x when we compute the likelihood for the ongoing study data $\tilde{x}$ . The left-truncated likelihood has an additional term in the denominator that renormalizes the truncated distribution so that it integrates to 1, that is,

(4) $$\mathrm{Left-truncated}\;\mathrm{likelihood}\;p_{LT}(\tilde{x}|\theta )=\prod_{i=1}^{n_{2}}\frac{h{\left({\tilde{x}}_{i},\theta \right)}^{{\tilde{\delta }}_{i}}S({\tilde{x}}_{i},\theta )}{S(t_{1},\theta )}.$$

Once we have derived the posterior distribution for the model parameters given data at $t_{1}$ , p(θ|x) , and the likelihood for the ongoing follow-up data, $p_{\mathrm{LT}}(\tilde{x}|\theta )$ , we require a method for actually computing expression (2). In almost all realistic applications, this will require numerical methods. Nested Monte Carlo can be used, but this is computationally expensive. A regression-based approach is much quicker,^10^ and this is described along with the Monte Carlo approach in Appendix B.

We are now in a position to describe methods for computing EVSI that account for uncertainty about the choice of survival model.

### Model-Averaged EVSI for an Ongoing Study Accounting for Survival Model Uncertainty

#### Survival model uncertainty and model averaging

In this section, “model” refers to the survival model for the time-to-event data p(x|θ) , not the cost-effectiveness model, NB(d,θ) . In many real applications, we will be uncertain about which survival model is most appropriate and should be used to extrapolate the data beyond the observed follow-up period $t_{1}$ , although we may be comfortable with proposing a candidate set of models, $M=M_{r},\;r=1,\ldots ,R$ , that covers plausible approximations of the data-generating process, that is, the set is M−open in the terminology used by Bernardo and Smith.^17^ In these circumstances, we may account for model uncertainty using predictive model averaging and average over model predictions using model weights based on each model’s predictive ability.^18,19^ After observing data x at time $t_{1}$ , we place probability weight $P(M_{r}|x)$ on the $r^{\mathrm{th}}$ model producing the best predictions, with $\sum_{r=1}^{R}P(M_{r}|x)=1$ .

The net benefit function for decision option d given model $M_{r}$ and parameters $\theta_{r}$ is denoted $\mathrm{NB}(d,\theta_{r},M_{r})$ . Taking the expectation over both parameters and models after observing data x up to time point $t_{1}$ gives us

(5) $$\begin{array}{cc} \mathrm{Model}-\mathrm{averaged}\;NB_{d}|x & =\sum_{r=1}^{R}\left\{E_{\theta_{r}|x,M_{r}}\mathrm{NB}(d,\theta_{r},M_{r})P(M_{r}|x)\right\}\\ =E_{M|x}[E_{\theta_{r}|x,M_{r}}\{\mathrm{NB}(d,\theta_{r},M_{r})\}]\\ =E_{\theta_{r},M|x}\{\mathrm{NB}(d,\theta_{r},M_{r})\}, \end{array}$$

and the optimal choice at time point $t_{1}$ is the decision d that maximizes this expectation.

#### EVSI for an ongoing study accounting for model uncertainty

Additional follow-up data $\tilde{x}$ will not only update our judgments about parameters, $p(\theta_{r}|x,\tilde{x},M_{r})$ , but will also update our judgments about the relative plausibility of each model, $P(M_{r}|x,\tilde{x})$ , for each model r=1,…,R .

The EVSI for an ongoing study, where we average over models, is given by

(6) $$\mathrm{Model-averaged}\;\;EVSI\;\;=\;\;E{\;}_{\tilde{x}|x}[\underset{d}{\max}\;E{\;}_{\theta_{r},ℳ|x,\tilde{x}}\{\;\mathrm{NB}\;(d,\theta_{r},M_{r})\}]-\underset{d}{\max}\;E{\;}_{\theta_{r},ℳ|x}\{\;\mathrm{NB}\;(d,\theta_{r},M_{r})\},$$

which is identical to equation (2), except that expectations are now taken over models as well as parameters (see Appendix C for a derivation).

To compute equation (6) we will need a method for generating plausible data sets $\tilde{x}$ from $p(\tilde{x}|x)$ , the distribution of the follow-up data given the observed data, which takes account of the fact that we now consider plausible a number of different data-generating models. We will also need to define model probabilities given observed data, $P(M_{r}|x)$ , and then find a method for computing posterior model probabilities $P(M_{r}|x,\tilde{x})$ , given each sampled future plausible data set $\tilde{x}$ . We address the issue of defining model probabilities given observed data first.

#### Deriving model probabilities given observed data up until $t_{1}$

We assume that before we see the observed data x , that we are indifferent about the “correct” model, so $P(M_{r})=1/1RR$ for all r . After we observe data x , we use the Akaike’s Information Criterion (AIC)^20^ to derive posterior model probabilities giving greater weight to models with better predictive ability (according to Kullback-Leibler divergence), as described by Jackson et al.^18^ We set

(7) $$P(M_{r}|x)=\frac{\exp\{-0.5\;\mathrm{AI}C_{r}(x)\}}{\sum_{r=1}^{R}\exp\{-0.5\;\mathrm{AI}C_{r}(x)\}},$$

where

$$\mathrm{AI}C_{r}(x)=-2\log\{p(x|{\hat{\theta }}_{r})\}+2u_{r}.$$

The term ${\hat{\theta }}_{r}$ is the maximum likelihood estimate for the parameters of model $M_{r}$ , and $u_{r}$ is the number of parameters in model $M_{r}$ .

#### Generating plausible ongoing follow-up data sets, $\tilde{x}$ , that we may observe between $t_{1}$ and $t_{2}$

Plausible data sets from the distribution $p(\tilde{x}|x)$ are generated as follows. First, we sample a model $M_{r}^{\left(k\right)}$ with probability $P(M_{r}|x)$ given by equation (7). Next, we draw a sample $\theta_{r}^{\left(k\right)}$ from the distribution of the parameters of our chosen model $p(\theta_{r}|x,M_{r}^{\left(k\right)})$ . Finally, we generate a data set ${\tilde{x}}^{\left(k\right)}$ from the distribution of the data $p(\tilde{x}|\theta_{r}^{\left(k\right)},M_{r}^{\left(k\right)})$ given the sampled parameter values $\theta_{r}^{\left(k\right)}$ and model $M_{r}^{\left(k\right)}$ . We can repeat this process k=1,…,K times to generate an arbitrary number of data sets.

#### Updating model probabilities given ongoing follow-up data from $t_{1}$ to $t_{2}$

We can derive our posterior model probabilities at time point $t_{2}$ , for data set ${\tilde{x}}^{\left(k\right)}$ , via Bayes theorem:

(8) $$P(M_{r}|x,{\tilde{x}}^{\left(k\right)})=\frac{p({\tilde{x}}^{\left(k\right)}|M_{r},x)P(M_{r}|x)}{\sum_{r=1}^{R}p({\tilde{x}}^{\left(k\right)}|M_{r},x)P(M_{r}|x)},$$

where $p({\tilde{x}}^{\left(k\right)}|M_{r},x)$ is the marginal likelihood (“marginal” because we have integrated out the model parameters):

$$p({\tilde{x}}^{\left(k\right)}|M_{r},x)=\int_{\Theta }p({\tilde{x}}^{\left(k\right)}|M_{r},\theta_{r})p(\theta_{r}|M_{r},x)d\theta_{r}.$$

We use bridge sampling to approximate the marginal likelihood, which is a form of importance sampling that has been shown to give good approximations in a wide range of settings.^21–24^ The key notion behind bridge sampling is that the marginal likelihood can be written as the ratio of 2 expectations, each of which can be estimated via importance sampling. The name “bridge” reflects the incorporation in the estimator of a density function that “bridges” (i.e., has good overlap with) the 2 densities from which samples are drawn. A detailed tutorial on the bridge sampling method is given in the article by Gronau et al.,^23^ and the method is straightforward to implement in the R package bridgesampling.^25^ Given the bridge sampling estimates of $p({\tilde{x}}^{\left(k\right)}|M_{r},x)$ for each model, posterior model probabilities are trivial to compute via expression (8).

As with single-model EVSI, computing model-averaged EVSI (expression [6]) will require numerical methods. Nested Monte Carlo and a regression-based approach are described in Appendix D. In the next section, we will apply these methods in a synthetic case study.

#### Synthetic case study

We will model survival with and without accounting for survival model uncertainty.

#### Decision problem and model definition

Our decision problem is to determine which of 2 treatment options has the longest mean survival: a new treatment (d=1) or standard care (d=2) .

In the single-model case, survival is assumed to follow a Weibull distribution, and the net benefit of each treatment option is assumed to equal the restricted mean survival time, given an overall time horizon of $t_{h}=240$ mo (i.e., the area under the survival curve from 0 to 240 mo). So the net benefit function is:

(9) $$\mathrm{NB}(d,\theta_{d})=\int_{0}^{t_{h}}\exp\left\{-{\left(\frac{t}{e^{\theta_{\lambda d}}}\right)}^{e^{\theta_{\mathrm{kd}}}}\right\}dt,$$

where the model parameters are the log-transformed Weibull shape and scale parameters, $\theta_{d}=(\theta_{\mathrm{kd}},\theta_{\lambda d})$ . Computing the restricted mean survival for distributions other than the exponential requires numerical integration, but easy-to-use functions are available in the R package flexsurv.^26^

In the model-averaged case, the decision problem is as above, but we assume we are uncertain about the choice of survival model, $M_{r}$ , to extrapolate the observed data beyond the current follow-up period $t_{1}$ . We assume that our set of plausible models M contains the following 4 parametric distributions: Weibull (r=1) , Gamma (r=2) , log-normal (r=3) , and log-logistic (r=4) .

#### Generating synthetic case study data sets, x , collected up to $t_{1}=12$ months

We generated 2 synthetic case study data sets: one in which the hazard of death is monotonically increasing and the other in which it is monotonically decreasing. For each case study, we generated a data set with 200 participants per trial arm with a maximum follow-up of $t_{1}=12$ mo. We denote the data sets $x_{1}$ for new treatment and $x_{2}$ for standard care.

To explore the performance of the method when the survival model was misspecified, we generated survival times evenly spaced from either a Weibull or a Gamma distribution, using the $0.005^{\mathrm{th}},0.015^{\mathrm{th}},\ldots ,0.985^{\mathrm{th}},0.995^{\mathrm{th}}$ quantiles from each distribution (i.e., 100 evenly spaced quantiles that avoid 0 and 1). We could have randomly generated survival times, but this would have just added additional Monte Carlo error when assessing the methods for computing EVSI. The parameters of the Weibull and Gamma distributions that we used to generate the synthetic case study data sets are shown in Table 2.

**Table 2.** Weibull and Gamma distribution parameters for the synthetic case study datasets

	Increasing Hazard Case Study		Decreasing Hazard Case Study	
	New Treatment	Standard Care	New Treatment	Standard Care
Weibull shape, k	1.10	1.10	0.60	0.60
Weibull scale, λ	70.00	50.00	80.00	57.00
Gamma shape, α	1.80	1.80	0.80	0.80
Gamma rate, β	0.04	0.04	0.01	0.01

**Figure 1:**
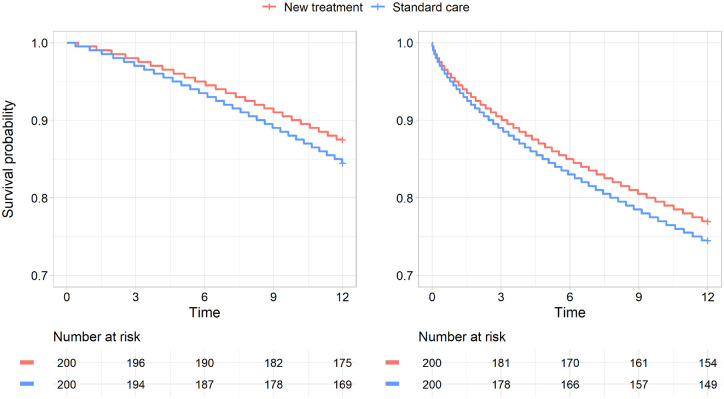
Kaplan-Meier plots for the increasing hazard data set (left) and decreasing hazard data set (right).

We enrolled all patients in the trial at $t_{0}=0$ and right censored the data sets at $t_{1}=$ 12 mo. We assumed no loss to follow-up and did not apply any other censoring. Figure 1 shows the Kaplan-Meier plots for the 2 synthetic case study data sets.

### Initial Trial Analysis at $t_{1}=12$ Months

For each synthetic case study, we analyzed the 2 trial arms separately. We fitted all 4 models to the data from each arm and estimated the model parameters using maximum likelihood (as implemented in the flexsurvreg function).^26^ We assumed that our judgments about the log-transformed parameters for each survival model conditional on the observed data up to $t_{1}$ , $p(\theta_{r}|x)$ , are represented by a bivariate normal distribution with the mean vector and covariance matrix derived from the maximum likelihood estimation. We computed the AIC for each model fit and derived model probability weights via equation (7).

Net benefits, AICs, and model probabilities are shown in Table 3, and means and covariances for each model are reported in Appendix G.

**Table 3:** Mean Survival, Akaike’s Information Criterion (AIC), and Prior Model Probabilities $P(M_{r}|x)$ for the 2 Hypothetical Data Sets

	Increasing Hazard Data Set			Decreasing Hazard Data Set		
	Net Benefit (Mean Survival)	AIC(x)	$P(M_{r}|x)$	Net Benefit (Mean Survival)	AIC(x)	$P(M_{r}|x)$
New treatment						
Weibull	50.96	277.58	0.26	84.81	437.06	0.29
Gamma	57.71	277.57	0.26	74.41	437.08	0.29
Log-normal	110.43	277.97	0.22	123.49	438.45	0.14
Log-logistic	79.28	277.58	0.26	105.98	437.14	0.28
Weighted average	72.93			93.31		
Standard care						
Weibull	44.01	329.26	0.28	77.85	470.37	0.30
Gamma	49.42	329.29	0.28	66.99	470.40	0.29
Log-normal	98.43	330.18	0.18	116.25	472.01	0.13
Log-logistic	71.00	329.33	0.27	99.70	470.47	0.28
Weighted average	62.36			85.85		
Incremental values						
Weighted average	10.57			7.46		

The expected net benefits (mean survival times) assuming a single Weibull model computed via Equation (9) are 50.96 versus 44.01 mo (incremental = 6.95 mo) for the increasing hazard data set and 84.81 versus 77.85 mo (incremental = 6.97 mo) for the decreasing hazard data set. The expected value of perfect information (EVPI) values, computed via Monte Carlo simulation with a sample size of $10^{5}$ , are 4.93 and 6.33 mo for the increasing and decreasing hazard data set, respectively.

The model-averaged net benefits, weighted by model probabilities, were 72.93 versus 62.36 mo (incremental = 10.57 mo) for the increasing hazard data set and 93.31 versus 85.85 mo (incremental = 7.46 mo) for the decreasing hazard dat set. The model-averaged EVPI values are 10.32 and 9.97 mo for the respective data sets.

#### Generating plausible ongoing follow-up data sets, $\tilde{x}$ , for the EVSI computation

Both the nested Monte Carlo and regression-based EVSI methods require a set of sampled ongoing follow-up data sets for each trial arm, denoted ${\tilde{x}}_{1}$ and ${\tilde{x}}_{2}$ . We generated k=1,…,K data sets with K= 6000 for each trial arm, where the $k^{\mathrm{th}}$ data set was generated as follows.

In the single-model case, we first sampled log-shape and log-scale values ( $\theta_{1}^{\left(k\right)}$ for new treatment and $\theta_{2}^{\left(k\right)}$ for standard care) from the bivariate normal distributions in Appendix G. We computed the net benefit for each decision option, given the sampled parameters, $\mathrm{NB}(d,\theta_{d}^{\left(k\right)})$ and stored this (these values are required for the regression-based approximation). For each arm, we then sampled n survival times from a truncated Weibull distribution (see Appendix E) with the sampled shape and scale values, where n was the number of patients who were still alive in the trial arm at $t_{1}=12$ mo. Finally, survival times were censored at the proposed endpoint for the ongoing data collection, $t_{2}$ .

In the model-averaged case, we first chose a model $M_{r}^{\left(k\right)}$ with probability $P(M_{r}|x)$ , before sampling $\theta_{r}^{\left(k\right)}$ from the bivariate normal distribution $p(\theta_{r}|x)$ for the chosen model $M_{r}^{\left(k\right)}$ and generating the n survival times for each arm. The remainder of the data-generation step is as above.

#### Computing EVSI for ongoing follow-up via nested Monte Carlo

To sample from the posterior distributions, $p(\theta_{d}|x_{d},{\tilde{x}}_{d}^{\left(k\right)})$ , we used Hamiltonian Monte Carlo (HMC) as implemented in the package rstan.^27^ HMC is a Metropolis-Hastings MCMC algorithm with a particularly efficient sampling scheme that reduces Monte Carlo sampling error, therefore requiring fewer posterior samples for any inference. The package rstan is an R interface to the Stan language.^28^ An alternative option would have been to use OpenBUGS.^29^

In the single-model case, for each outer loop sampled data set, k=1,…, 6000, we averaged the net benefit functions over J= 2000 inner loop posterior samples of the model parameters and stored the maximum net benefit of the 2 treatment options. We then averaged these maximized net benefits and subtracted the expected value of a decision based on current information to obtain the EVSI following expression (5) in Appendix B.

In the model-averaged case, for each outer loop data set, we generated the J posterior samples of the model parameters for each of the r=1,…,4 models (we needed to identify the truncated likelihood function for each model as we did for the Weibull example above, but this is straightforward (see Appendix E). We weighted the parameter averaged net benefits ${\mathrm{NB}}_{r}^{k}(d)$ by the posterior model probabilities $P(M_{r}|{\tilde{x}}^{\left(k\right)})$ to give the posterior model-averaged expected net benefit and identified the treatment d that maximized this for iteration k=1,…, 6000. We then subtracted the expected value of a decision based on current information to obtain the EVSI following expression (14) in Appendix D.

#### Computing EVSI for ongoing follow-up via regression

The generalized additive model (GAM) approach to computing EVSI for extending the follow-up until time $t_{2}$ for the hypothetical example is as follows.

For each trial arm, we computed a low-dimensional summary statistic for each data set. A convenient choice here is the number of observed events $e_{d}^{\left(k\right)}$ and the total time at risk $y_{d}^{\left(k\right)}$ for each data set ${\tilde{x}}_{d}^{\left(k\right)}$ , that is, $T({\tilde{x}}_{d}^{\left(k\right)})=\{e_{d}^{\left(k\right)},y_{d}^{\left(k\right)}\}$ for d=1,2 .

Then, for each of the 2 decision options, we fitted a GAM regression model with the stored net benefits $\mathrm{NB}(d,\theta_{d}^{\left(k\right)})$ as the dependent variable and the two summary statistics, $e_{d}^{\left(k\right)}$ and $y_{d}^{\left(k\right)}$ , as independent variables. We allowed a smooth, arbitrary, nonlinear relationship between the independent and dependent variables, plus an arbitrary interaction between the independent variables, by specifying a tensor product cubic regression spline basis for the independent variables. This has the simple syntax gam(nb_d ∼ te(e_d, y_d)) in the mgcv^30^ package in R. We extracted the GAM model fitted values ${\hat{g}}_{d}^{\left(k\right)}$ from each regression model fit and estimated the EVSI using equation (9) in Appendix B.

The GAM-based approximation method for model-averaged EVSI is identical to that used in the single-model case.

## Results

### EVSI Values for the Weibull Ongoing Data

The nested Monte Carlo– and GAM-based EVSI estimates for additional follow-up times of 12, 24, 36, and 48 mo (i.e., $t_{2}=24,36,48,60$ mo) are shown in Table 4. The methods used to estimate the standard errors of the nested Monte Carlo and GAM estimators are described in an appendix of the article by Strong et al.^31^

**Table 4:** EVSI (SE) Values for Additional Follow-up Time for the 2 Hypothetical Data Sets Given a Weibull Distribution for the Survival Times

Additional Follow-up (mo)	Increasing Hazard Data Set		Decreasing Hazard Data Set	
	Nested Monte Carlo	GAM	Nested Monte Carlo	GAM
12	4.25 (0.09)	4.28 (0.08)	4.41 (0.10)	4.46 (0.10)
24	4.58 (0.09)	4.62 (0.06)	5.20 (0.11)	5.27 (0.09)
36	4.68 (0.09)	4.71 (0.05)	5.45 (0.11)	5.54 (0.08)
48	4.74 (0.09)	4.77 (0.04)	5.55 (0.11)	5.65 (0.07)

EVSI, expected value of sample information; GAM, generalized additive model.

a EVPI values are 4.93 and 6.33, respectively. Total computation times for the analyses in the table are 24,808 s (nested Monte Carlo) and 36 s (GAM).

As expected, the EVSI reflects the diminishing marginal returns for increasing the follow-up duration and converges toward the EVPI. The EVSI varies depending on the underlying hazard pattern, even when point estimates of mean incremental survival benefit are similar (6.95 mo for the increasing hazard data set and 6.97 mo for the decreasing hazard data set). The increasing hazard data set has lower numbers of prior observed events and higher expected numbers of future events for the additional follow-up time than the decreasing hazard data set does, which—all else equal—is expected to result in greater EVSI values. This upward effect on EVSI is, however, canceled out by the downward effect of lower estimates of mean survival, resulting in greater EVSI values for the decreasing hazard data set than for the increasing hazard data set.

The GAM method agrees well with the MCMC method, with the benefit of a greatly reduced computational cost. The MCMC inner loop for the Monte Carlo method used parallel processing, but even with this additional efficiency, the regression method was approximately 700 times faster than the nested Monte Carlo method was. We used a machine running Windows 10 with an Intel Core i9 CPU with 15 threads running on 8 cores at 2.40 GHz and with 32 GB RAM.

Of note is that the standard errors for the nested Monte Carlo estimator slightly increase with increasing follow-up duration, while the opposite is true for the GAM estimator. This is due to different mechanisms through which the effective sample size of the generated data $\tilde{x}$ affects the standard errors of the nested Monte Carlo and GAM estimators, which is further explained in Appendix F.

### Model-Averaged EVSI Values

The nested Monte Carlo– and GAM-based model-averaged EVSI estimates for additional follow-up times of 12, 24, 36, and 48 mo (i.e., $t_{2}=24,36,48,60$ mo) are shown in Table 5.

**Table 5:** Model-averaged EVSI (SE) Values for Additional Follow-up Time for the 2 Hypothetical Data Sets Given a Mixture of Weibull, Gamma, Lognormal and Log-logistic Distributions for the Survival Times

Additional Follow-up (mo)	Increasing Hazard Data Set		Decreasing Hazard Data Set	
	Nested Monte Carlo	GAM	Nested Monte Carlo	GAM
12	7.50 (0.18)	7.52 (0.14)	6.69 (0.15)	6.70 (0.13)
24	8.75 (0.20)	8.82 (0.10)	8.09 (0.18)	8.16 (0.11)
36	9.43 (0.21)	9.44 (0.08)	8.71 (0.19)	8.76 (0.09)
48	9.77 (0.22)	9.74 (0.07)	8.96 (0.19)	9.01 (0.08)

EVSI, expected value of sample information; GAM, generalized additive model.

a EVPI values are 10.32 and 9.97, respectively. Total computation times for the analyses in the table are 289,211 s (nested Monte Carlo) and 37 s (GAM).

As expected, the EVSI converges toward the EVPI as follow-up time increases, and there is good agreement between the 2 methods. The model-averaged EVSI values for additional follow-up are greater than the Weibull model EVSI (Table 4), which reflects the additional value in reducing model as well as parameter uncertainty. The GAM method is approximately 8000 times faster than the nested Monte Carlo method.

#### Expected net benefit of sampling

The net value of additional data collection can be quantified by computing the expected net benefit of sampling (ENBS).^32^ In the context of an ongoing study, the ENBS is the difference between the EVSI for collecting additional data between $t_{1}$ and $t_{2}$ and the expected cost of continuing the study and potential health benefits foregone if approval is withheld. When the ENBS is positive, it is worthwhile to continue the study and collect more data before making an adoption decision.

If the adoption decision is reversible, then there are 2 decision options given that the new technology is expected to improve net health benefits: “approval with research” (AWR), which refers to approval while additional data are being collected, or “only in research” (OIR), which means a decision to approve or reject is withheld until additional data have been collected.^6^ An adoption decision may also be reversible with a cost, in which case the EVSI for AWR will be lower than for OIR.^7^ For example, some irrecoverable costs, such as high initial treatment costs that are offset only by later health benefits, may be avoided if treatment initiation could be delayed until additional data have been collected.^33^ If these avoidable costs are large, OIR may potentially be more appropriate than AWR, even if the decision is reversible. OIR may also be recommended if the new technology is not expected to improve net health benefits, but there is value in collecting additional data. If the adoption decision is irreversible, or approval would mean that further research could not be conducted, then AWR is not available and OIR may be the only option. In these circumstances, opportunity costs, in terms of potential net health benefits foregone, will be incurred while the research is being conducted if the new technology is expected to improve net health benefits.

Establishing population ENBS requires an assessment of the number of current and future patients who may benefit from additional data collection over the decision relevance time horizon.^34^ The cost of continuing an ongoing study will primarily consist of variable (per patient) costs, including marginal incremental treatment costs and marginal reporting costs. Fixed study costs that have already been incurred will not affect the decision to continue the ongoing study or not.

**Figure 2:**
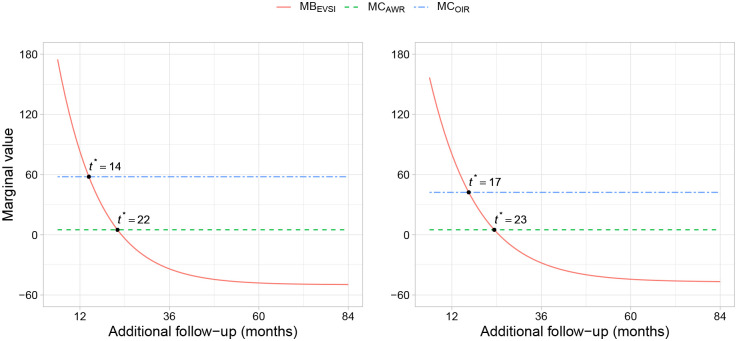
Marginal benefit ( $MB_{\mathrm{EVSI}}$ ), marginal cost of “approval with research” ( $MC_{\mathrm{AWR}}$ ) and, marginal cost of “only in research” ( $MC_{\mathrm{OIR}}$ ) given different durations of additional follow-up. Estimates are based on the model-averaged EVSI analyses for the increasing hazard data set (left) and decreasing hazard data set (right), trial costs of 5 life-months per month, 5 new patients receiving treatment each month, and a decision time horizon of 10 y.

Figure 2 illustrates that if AWR is recommended, the marginal benefit in terms of population model-averaged EVSI equals the marginal cost of continuing the trial at 22 and 23 mo of additional follow-up for the increasing and decreasing hazard data sets, respectively. These are the time points at which the ENBS is at a maximum. If OIR is recommended, the ENBS is at a maximum when the marginal benefit of delaying the decision until more data have been collected equals the marginal cost of continuing the trial and withholding approval, which is at 14 and 17 mo of additional follow-up for the increasing and decreasing hazard data sets, respectively.

## Discussion

EVSI is useful not only for informing the design of a future trial but also for deciding whether an ongoing study should continue in order to collect additional data before making an adoption decision. This article is the first to set out generic EVSI algorithms for survival data from an ongoing trial with or without accounting for survival model uncertainty. The EVSI algorithms generalize to any decision context in which structural uncertainty is present, provided that the analyst is able to derive probability weights for the competing scenarios.

### Strengths and Limitations

The nonparametric regression-based method is fast and straightforward to implement, even when we include consideration of model uncertainty. In fact, extending the method to include model uncertainty does not increase the complexity or computation time. The nested Monte Carlo procedure, on the other hand, is extremely computationally demanding when we include model uncertainty.

Although we considered only 2 treatment options in the synthetic case study, the EVSI methods described in this article extend to any number of treatment options that are being compared. This requires the generation of data for each treatment arm for which additional data will be collected conditional on samples from the distribution of the model parameters, which could be drawn from independently fitted survival models or from a joint model based on proportional hazards or an accelerated failure time assumption. The net benefit for each treatment arm can then be regressed on the treatment arm–specific summary statistics of the generated data, and EVSI can be computed the usual way following the algorithms in this article.

When a large part of the relevant time horizon is unobserved, the clinical plausibility of the survival extrapolations is often of greater importance than the mathematical fit to the observed data.^14^ Deriving prior model probabilities from purely statistical measures such as AIC may therefore not always be appropriate when data are immature, since these measures do not reflect the plausibility of the extrapolations.^8^ This became evident in the synthetic case studies, as the AIC-based prior model probabilities of the log-normal and log-logistic models were similar to those of the Weibull and Gamma models for the increasing hazard data set, despite the fact that the former 2 models do not allow for monotonically increasing hazards and therefore cannot capture the true underlying hazard pattern.

Similar to previous work,^9,35^ we have viewed model uncertainty in terms of a discrete model space, which can be addressed by model averaging. An alternative view on model uncertainty could involve indexing candidate models within a continuous model space, using a single very flexible model that includes all the models the analyst believes plausible. For example, the generalized F distribution includes most commonly used parametric survival distributions as special cases.^36^ In this case, the EVSI algorithms in this article would reduce to the single-model case. This approach, however, requires the specification of a prior that appropriately reflects uncertainty in choosing between alternative functional forms within the flexible model, which may be not be straightforward. Flexible models such as the generalized F distribution, Royston-Parmar spline models, or fractional polynomials are also prone to overfitting and may not always provide reliable predictions of mean survival, particularly when data are immature.^9^

For the purposes of describing the new method, we assumed that the survival distribution in the future unobserved time period is the same as in the observed period. This is a simplifying assumption that may not hold in real-life settings. Most importantly, we may have additional uncertainty about the postobservation period that is not captured by the uncertainty encoded in the survival model probability distribution. For example, the duration of the treatment effect is conditional on multiple factors such as the biological effect mechanism, treatment-stopping rules, compliance, and side effects.^37^ The extrapolation of trial data may therefore have to be supplemented with external evidence^38^ and assumptions about disease progression and mechanisms of action of the treatments that reflects additional knowledge and uncertainty. This typically involves eliciting expert opinion.^39^ We also did not consider flexible parametric models such as Royston-Parmar spline-based models^40^ or mixture cure models^41^ in the synthetic case studies. Although the EVSI methods described in this article apply equally to any survival distribution and underlying assumptions (including those regarding the duration of the treatment effect), they require the generation of plausible data sets that obey all the model rules, which may not be straightforward for complex study designs. This is a common limitation of existing EVSI methods, and more research in this area may be needed.

In the synthetic case studies, we assumed all patients had the same follow-up at $t_{1}$ . In clinical trials, patients are usually recruited over a period of time, which means the individual follow-up times will vary at $t_{1}$ . In these circumstances, additional follow-up will provide more information not only about the tail of the survival curve (from patients who were enrolled early) but also about the central part (from patients who were enrolled later).

We did not consider sequential trial designs,^42^ which require EVSI to be recalculated after each observation and to account for all the possible ways in which future patients may be allocated to the trial arms or when to stop the trial.^34^ This can give rise to a large number of subproblems that may have to be solved using dynamic programming methods, which can be computationally very demanding.

### Policy Implications

Immature evidence leads to a high level of decision uncertainty, which may result in the uptake of technologies that reduce net health benefit. The decision-making context in which trials are ongoing and evidence is immature is particularly pronounced for new oncology drugs. The purpose of the Cancer Drug Fund (CDF) in the United Kingdom, for example, is to enable early patient access to promising new cancer drugs while allowing evidential uncertainty to be reduced through ongoing data collection. In the period between 2017 and July 2018, the National Institute for Health and Care Excellence (NICE) recommended more than half of the appraised cancer drugs through the CDF, typically because of concerns about immature survival data.^43^

EVSI will depend on both the study design and the decision context^44,45^ but also on whether the trial results generalize to multiple jurisdictions^46,47^ and whether the adoption decision can be fully implemented.^48,49^ The benefit of additional data collection can be realized only when trial results are reported.^50^ An assessment is therefore required of when the ongoing trial might report and at which point the adoption decision can be revisited.^51^ Risk-sharing agreements between a manufacturer and payer may potentially modify the value of collecting additional data as well as the expected net benefit of access to a new technology.^6,47,52^ The option to enroll more patients into an ongoing trial should also be considered if it has a positive net value.

The EVSI algorithms in this article can help decision makers determine whether early patient access to a new technology can be justified on the basis of the current evidence or whether more mature evidence is needed. Unlike most of the existing work on EVSI that primarily targets commissioners and funders of research, EVSI for ongoing trials also addresses the policy context of decision makers who do not have the remit to commission additional research.

## Supplemental Material

sj-pdf-1-mdm-10.1177_0272989X211068019 – Supplemental material for An Efficient Method for Computing Expected Value of Sample Information for Survival Data from an Ongoing Trial Click here for additional data file. Supplemental material, sj-pdf-1-mdm-10.1177_0272989X211068019 for An Efficient Method for Computing Expected Value of Sample Information for Survival Data from an Ongoing Trial by Mathyn Vervaart, Mark Strong, Karl P. Claxton, Nicky J. Welton, Torbjørn Wisløff and Eline Aas in Medical Decision Making
