# Supplementary material for: An Efficient Method for Computing Expected Value of Sample Information for Survival Data from an Ongoing Trial
Source: Med Decis Making. 2021 Dec 30;42(5):612–25. doi: 10.1177/0272989X211068019 (PMC9189722; doi:10.1177/0272989X211068019)
Supplement: sj-pdf-1-mdm-10.1177_0272989X211068019 – Supplemental material for An Efficient Method for Computing Expected Value of Sample Information for Survival Data from an Ongoing Trial [file sj-pdf-1-mdm-10.1177_0272989X211068019.pdf]

An Efficient Method for Computing Expected Value of Sample Information for Survival Data from an Ongoing Trial. Supplementary online material.

Mathyn Vervaart[1,2,*], Mark Strong[3], Karl P. Claxton[4,5], Nicky J. Welton[6], Torbjørn Wisløff[7,8], Eline Aas[1]

[1]Department of Health Management and Health Economics, University of Oslo, Oslo, Norway

[2]Norwegian Medicines Agency, Oslo, Norway

[3]School of Health and Related Research, University of Sheffield, Sheffield, UK

[4]Centre for Health Economics, University of York, York, UK

[5]Department of Economics and Related Studies, University of York, York, UK

[6]Population Health Sciences, University of Bristol, Bristol, UK

[7]Department of Community Medicine, UiT The Arctic University of Norway, Oslo, Norway

[8]Norwegian Institute of Public Health, Oslo, Norway

Appendix A - Deriving an expression for the EVSI for an ongoing study assuming no model uncertainty

Before the study starts, we have only prior knowledge about model parameters, which we represent via the distribution $p(\boldsymbol{\theta})$. In many cases we will not have strong prior information, and $p(\boldsymbol{\theta})$ will therefore be minimally informative (typically flat on some scale).

We collect data $\mathbf{x}$ during an initial period of follow-up that extends up until time t_1. At t_1 we update our judgements about $\boldsymbol{\theta}$, conditional on $\mathbf{x}$ to give the posterior distribution $p(\boldsymbol{\theta}|\mathbf{x})$. The question is, should we continue the study to collect more data before making an adoption decision?

The optimal decision option given observed data up to time t_1 has expected value

$$\max_d \mathbb{E}_{\boldsymbol{\theta}|\mathbf{x}}\{\text{NB}(d,\boldsymbol{\theta})\}. \tag{1}$$

Further data collection up until time point t_2 will give us additional data $\tilde{\mathbf{x}}$, which we can use to update judgements about θ to give $p(\boldsymbol{\theta}|\mathbf{x},\tilde{\mathbf{x}})$. The optimum decision option will have expected value,

$$\max_d \mathbb{E}_{\boldsymbol{\theta}|\mathbf{x},\tilde{\mathbf{x}}}\{\text{NB}(d,\boldsymbol{\theta})\}. \tag{2}$$

At time point t_1 data $\tilde{\mathbf{x}}$ are as yet uncollected, however we can take the expectation of expression (2) with respect to the distribution of the ongoing follow-up data $\tilde{\mathbf{x}}$ conditional on the observed follow-up data $\mathbf{x}$, $p(\tilde{\mathbf{x}}|\mathbf{x})$, giving

$$\mathbb{E}_{\tilde{\mathbf{x}}|\mathbf{x}}[\max_d \mathbb{E}_{\boldsymbol{\theta}|\mathbf{x},\tilde{\mathbf{x}}}\{\text{NB}(d,\boldsymbol{\theta})\}]. \tag{3}$$

*Corresponding author: Mathyn Vervaart, Department of Health Management and Health Economics, University of Oslo, Forskningsveien 3A, Harald Schjelderups hus, 0373 Oslo, Norway (mathyn.vervaart@medisin.uio.no).

The EVSI for continuing the study from t_1 to t_2 is the difference between the expected value of a decision made after collecting data up to t_2, expression (3), and the expected value of a decision based on observed data collected up to t_1, expression (1),

$$\text{EVSI(ongoing study)} = \mathbb{E}_{\tilde{\mathbf{x}}|\mathbf{x}}[\max_d \mathbb{E}_{\boldsymbol{\theta}|\mathbf{x},\tilde{\mathbf{x}}}\{\text{NB}(d,\boldsymbol{\theta})\}] - \max_d \mathbb{E}_{\boldsymbol{\theta}|\mathbf{x}}\{\text{NB}(d,\boldsymbol{\theta})\}. \quad (4)$$

Appendix B - Methods for computing the EVSI for an ongoing study assuming no model uncertainty

Nested Monte Carlo method for computing EVSI for an ongoing study

Calculating EVSI for an ongoing study requires evaluation of the expectation of a maximised conditional expectation, $\mathbb{E}_{\tilde{\mathbf{x}}|\mathbf{x}}[\max_d \mathbb{E}_{\theta|\mathbf{x},\tilde{\mathbf{x}}}\{\text{NB}(d,\boldsymbol{\theta})\}]$. This will rarely have an analytic solution. A nested expectation can be evaluated using a nested 'double-loop' Monte Carlo scheme, which leads us to the following estimator for EVSI,

$$\text{EVSI} \simeq \frac{1}{K}\sum_{k=1}^{K}\max_d \frac{1}{J}\sum_{j=1}^{J}\text{NB}(d,\boldsymbol{\theta}^{(j,k)}) - \max_d \frac{1}{K}\sum_{k=1}^{K}\frac{1}{J}\sum_{j=1}^{J}\text{NB}(d,\boldsymbol{\theta}^{(j,k)}). \quad (5)$$

In this scheme, we generate samples from $p(\tilde{\mathbf{x}}|\mathbf{x})$ in the 'outer loop.' We do this by first sampling $\boldsymbol{\theta}^{(k)}$, $k = 1,\ldots,K$ from $p(\boldsymbol{\theta}|\mathbf{x})$, and then sampling $\tilde{\mathbf{x}}^{(k)}$ from the truncated likelihood $p_{LT}(\tilde{\mathbf{x}}|\boldsymbol{\theta}^{(k)})$. For each sample $\tilde{\mathbf{x}}^{(k)}$, we then sample values $\boldsymbol{\theta}^{(j,k)}$, $j = 1,\ldots,J$ from the posterior distribution $p(\boldsymbol{\theta}|\mathbf{x},\tilde{\mathbf{x}}^{(k)})$ in the 'inner loop'. Unless $p({}_{LT}\tilde{\mathbf{x}}|\boldsymbol{\theta})$ and $p(\boldsymbol{\theta}|\mathbf{x})$ are conjugate, which will be rare in practice, then sampling from $p(\boldsymbol{\theta}|\mathbf{x},\tilde{\mathbf{x}}^{(k)})$ will require Markov Chain Monte Carlo (MCMC) or a similar scheme. The total number of samples required for each d is $J \times K$.

Note that the second term in expression (5) has a nested double loop structure, even though the target estimand is the single maximised expectation $\max_d \mathbb{E}_{\boldsymbol{\theta}|\mathbf{x}}\{\text{NB}(d,\boldsymbol{\theta})\}$. We reuse the same samples for both terms in the EVSI expression in order to reduce Monte Carlo error, noting that $\max_d \mathbb{E}_{\tilde{\mathbf{x}}|\mathbf{x}}[\mathbb{E}_{\boldsymbol{\theta}|\mathbf{x},\tilde{\mathbf{x}}}\{\text{NB}(d,\boldsymbol{\theta})\}] = \max_d \mathbb{E}_{\boldsymbol{\theta}|\mathbf{x}}\{\text{NB}(d,\boldsymbol{\theta})\}$ by the law of total expectation.[1]

Regression-based method for computing EVSI for an ongoing study

Strong and others (2015)[1] developed a fast, non-parametric regression-based method that greatly reduces the computational burden of the nested Monte Carlo procedure to EVSI. Their approach relies on estimating the functional relationship between the posterior expected net benefits and the generated datasets, thereby avoiding the inner loop and markedly increasing efficiency over the nested Monte Carlo method.

In the regression approach, we first generate a random parameter vector $\boldsymbol{\theta}^{(k)}$ from the distribution of model parameters $p(\boldsymbol{\theta}|\mathbf{x})$ at time point t_1, and a random data sample $\tilde{\mathbf{x}}^{(k)}$ from the truncated likelihood $p_{LT}(\tilde{\mathbf{x}}|\boldsymbol{\theta}^{(k)})$, where k indicates the k_{th} sample. The net benefit is evaluated at the same k^{th} sample of the model parameters, $\text{NB}(d,\boldsymbol{\theta}^{(k)})$. We then express the observed net benefit $\text{NB}(d,\boldsymbol{\theta}^{(k)})$ as a sum of the conditional expectation of the net benefit given the data, $\mathbb{E}_{\boldsymbol{\theta}|\mathbf{x},\tilde{\mathbf{x}}^{(k)}}\{\text{NB}(d,\boldsymbol{\theta})\}$, which we wish to estimate to evaluate the EVSI (Equation (4)), and a mean-zero error term, $\varepsilon^{(k)}$,

$$\text{NB}(d,\boldsymbol{\theta}^{(k)}) = \mathbb{E}_{\boldsymbol{\theta}|\mathbf{x},\tilde{\mathbf{x}}^{(k)}}\{\text{NB}(d,\boldsymbol{\theta})\} + \varepsilon^{(k)}. \quad (6)$$

As explained by Strong and others (2015)[1], we can think of the conditional expectation $\mathbb{E}_{\boldsymbol{\theta}|\mathbf{x},\tilde{\mathbf{x}}^{(k)}}\{\text{NB}(d,\boldsymbol{\theta})\}$ as an unknown function of $\tilde{\mathbf{x}}^{(k)}$. We denote this function $g(d,\tilde{\mathbf{x}}^{(k)})$ and substitute this into Equation (6), giving

$$\text{NB}(d,\boldsymbol{\theta}^{(k)}) = g(d,\tilde{\mathbf{x}}^{(k)}) + \varepsilon^{(k)}. \quad (7)$$

Since $\tilde{\mathbf{x}}$ is a vector of (possibly censored) time-to-event data, and therefore high-dimensional, we write the the

function g in terms of a low-dimensional summary statistic of the data $T(\tilde{\mathbf{x}})$,

$$\mathrm{NB}(d, \boldsymbol{\theta}^{(k)}) = g\{d, T(\tilde{\mathbf{x}}^{(k)})\} + \varepsilon^{(k)}. \tag{8}$$

We then use a generalized additive model (GAM), which is a flexible non-parametric regression model, to estimate the target function g. This means that we fit a GAM model to each decision option d and extract the regression model fitted values to estimate posterior net benefit. We denote the GAM model fitted values as $\hat{g}_d^{(k)}$. The GAM-based EVSI estimate is given by

$$\mathrm{EVSI} \simeq \frac{1}{K}\sum_{k=1}^{K} \max_d \hat{g}_d^{(k)} - \max_d \frac{1}{K}\sum_{k=1}^{K} \hat{g}_d^{(k)}. \tag{9}$$

Appendix C - Deriving an expression for the EVSI for an ongoing study accounting for model uncertainty

In the model averaging setting, additional follow-up data $\tilde{\mathbf{x}}$ will update our judgements about both parameters and the relative plausibility of each model.

The net benefit function for decision option d given model M_r and parameters $\boldsymbol{\theta}_r$ is denoted $\mathrm{NB}(d, \boldsymbol{\theta}_r, M_r)$. At time point t_1 after observing data $\mathbf{x}$, the expected net benefit, averaging over both parameters and models is

$$\begin{aligned}\text{Model-averaged } \mathrm{NB}_d|\mathbf{x} &= \sum_{r=1}^{R} \left\{\mathbb{E}_{\boldsymbol{\theta}_r|\mathbf{x},M_r} \mathrm{NB}(d, \boldsymbol{\theta}_r, M_r) P(M_r|\mathbf{x})\right\} \\ &= \mathbb{E}_{\mathcal{M}|\mathbf{x}}[\mathbb{E}_{\theta_r|\mathbf{x},M_r}\{\mathrm{NB}(d, \boldsymbol{\theta}_r, M_r)\}] \\ &= \mathbb{E}_{\boldsymbol{\theta}_r,\mathcal{M}|\mathbf{x}}\{\mathrm{NB}(d, \boldsymbol{\theta}_r, M_r)\},\end{aligned} \tag{10}$$

and the optimal choice at time point t_1 is the decision d that maximises this expectation.

The net benefit after observing ongoing follow-up data $\tilde{\mathbf{x}}$ between t_1 and t_2 is

$$\begin{aligned}\text{Model-averaged } \mathrm{NB}_d|\mathbf{x}, \tilde{\mathbf{x}} &= \sum_{r=1}^{m} \left\{\mathbb{E}_{\boldsymbol{\theta}_r|\mathbf{x},\tilde{\mathbf{x}},M_r} \mathrm{NB}(d, \boldsymbol{\theta}_r, M_r) P(M_r|\mathbf{x}, \tilde{\mathbf{x}})\right\} \\ &= \mathbb{E}_{\boldsymbol{\theta}_r,\mathcal{M}|\mathbf{x},\tilde{\mathbf{x}}}\{\mathrm{NB}(d, \boldsymbol{\theta}_r, M_r)\},\end{aligned} \tag{11}$$

and the optimal choice at time point t_2 is the decision d that maximises this expectation. Follow-up data $\tilde{\mathbf{x}}$ are not available at t_1, but we can compute the *expected value* of the maximised net benefit based on our beliefs from the data collected by t_1,

$$\mathbb{E}_{\tilde{\mathbf{x}}|\mathbf{x}}\left[\max_d \mathbb{E}_{\boldsymbol{\theta}_r,\mathcal{M}|\mathbf{x},\tilde{\mathbf{x}}}\{\mathrm{NB}(d, \boldsymbol{\theta}_r, M_r)\}\right]. \tag{12}$$

The EVSI for an ongoing study, where we average over models, is then the difference between (12) and the maximised value of (10),

$$\text{Model-averaged EVSI} = \mathbb{E}_{\tilde{\mathbf{x}}|\mathbf{x}}\Big[\max_d \mathbb{E}_{\boldsymbol{\theta}_r,\mathcal{M}|\mathbf{x},\tilde{\mathbf{x}}}\{\mathrm{NB}(d, \boldsymbol{\theta}_r, M_r)\}\Big] - \max_d \mathbb{E}_{\boldsymbol{\theta}_r,\mathcal{M}|\mathbf{x}}\{\mathrm{NB}(d, \boldsymbol{\theta}_r, M_r)\}. \tag{13}$$

Appendix D - Methods for computing model-averaged EVSI for an ongoing study

Nested Monte Carlo method for computing model-averaged EVSI

The nested double-loop Monte Carlo scheme in expression (5) naturally extends to the nested triple loop scheme when we average over models as well as over parameters and datasets,

$$\begin{aligned}\text{Model-averaged EVSI} \simeq & \frac{1}{K}\sum_{k=1}^{K}\max_d \sum_{r=1}^{R}\frac{1}{J}\sum_{j=1}^{J}\text{NB}(d,\boldsymbol{\theta}_r^{(j,k)},M_r^{(k)})P(M_r^{(k)}|\mathbf{x},\tilde{\mathbf{x}}^{(k)}) \\ & -\max_d \frac{1}{K}\sum_{k=1}^{K}\sum_{r=1}^{R}\frac{1}{J}\sum_{j=1}^{J}\text{NB}(d,\boldsymbol{\theta}_r^{(j,k)},M_r^{(k)})P(M_r^{(k)}|\mathbf{x},\tilde{\mathbf{x}}^{(k)}). \qquad (14)\end{aligned}$$

In this (somewhat intimidating looking) scheme, we first generate $k = 1,\ldots,K$ samples $\tilde{\mathbf{x}}^{(k)}$ from $p(\tilde{\mathbf{x}}|\mathbf{x})$ in the 'outer loop' (as described in the generating datasets section above). Then, in the inner loop, we compute posterior expected net benefits by drawing $j = 1,\ldots,J$ samples $\boldsymbol{\theta}_r^{(j,k)}$ from $p(\boldsymbol{\theta}_r|\mathbf{x},\tilde{\mathbf{x}}^{(k)},M_r^{(k)})$ and take the average for each treatment d. This inner loop sampling from the posterior distribution of the parameters typically requires MCMC, unless the prior and truncated likelihood are conjugate. Finally, for each k, we compute the posterior model probability $P(M_r^{(k)}|\mathbf{x},\tilde{\mathbf{x}}^{(k)})$ for each model $r = 1,\ldots,R$ (again, as described above).

As before, we reuse the same samples for both terms in the EVSI expression in order to reduce Monte Carlo error, noting that $\max_d \mathbb{E}_{\tilde{\mathbf{x}}|\mathbf{x}}(\mathbb{E}_{\mathcal{M}|\mathbf{x},\tilde{\mathbf{x}}}[\mathbb{E}_{\boldsymbol{\theta}_r|\mathbf{x},\tilde{\mathbf{x}},M_r}\{\text{NB}(d,\boldsymbol{\theta}_r,M_r)\}]) = \max_d \mathbb{E}_{\boldsymbol{\theta}_r,\mathcal{M}|\mathbf{x}}\{\text{NB}(d,\boldsymbol{\theta}_r,M_r)\}$ by the law of total expectation. The total number of samples required for each d is $J \times R \times K$.

The nested triple-loop Monte Carlo scheme for computing model-averaged EVSI is given in Box 1.

Box 1: Nested Monte Carlo Scheme for Computing Model-Averaged EVSI

for $k = 1,\ldots,K$ outer loops **do**
- Sample a model $M_r^{(k)}$ given current data $\mathbf{x}$ with probability $P(M_r|\mathbf{x})$
- Sample $\boldsymbol{\theta}_r^{(k)}$ from the distribution of the parameters of the sampled model, $p(\boldsymbol{\theta}_r|\mathbf{x},M_r^{(k)})$
- Generate a new data sample $\tilde{\mathbf{x}}^{(k)}$ from the distribution of the data $p(\tilde{\mathbf{x}}|\boldsymbol{\theta}_r^{(k)},M_r^{(k)})$
- **for** $r = 1,\ldots,R$ models **do**
  - Compute posterior expected net benefits by drawing $j = 1,\ldots,J$ inner loop samples $\boldsymbol{\theta}_r^{(j,k)}$ from $p(\boldsymbol{\theta}_r|\mathbf{x},\tilde{\mathbf{x}}^{(k)},M_r^{(k)})$ and take the average for each decision option d
- **end**
- Compute the posterior model probabilities $P(M_1^{(k)},\ldots,M_R^{(k)}|\mathbf{x},\tilde{\mathbf{x}}^{(k)})$
- Find the decision option d that maximises model-averaged posterior expected net benefit for iteration k

end

Compute the expected value of a decision based on new data $\tilde{\mathbf{x}}$ by taking the average of the maximum expected net benefits over the K iterations

Compute the expected value of a decision based on current data $\mathbf{x}$ by finding the decision option d that maximises the average of the expected net benefits over the K iterations

Compute the EVSI by subtracting the expected value of a decision based on current data from the expected value of a decision based on new data

Regression-based method for computing model-averaged EVSI

The non-parametric regression-based method for computing model-averaged EVSI is a natural extension of the regression-based method for a single known model described above. Firstly, we sample a model $M_r^{(k)}$ with probability $P(M_r|\mathbf{x})$. Next, we draw a sample $\boldsymbol{\theta}_r^{(k)}$ from the distribution of the parameters of our chosen

model $p(\boldsymbol{\theta}_r|\mathbf{x}, M_r^{(k)})$.

We then generate a dataset $\tilde{\mathbf{x}}^{(k)}$ from the distribution of the data $p(\tilde{\mathbf{x}}|\boldsymbol{\theta}_r^{(k)}, M_r^{(k)})$ given the sampled parameter values $\boldsymbol{\theta}_r^{(k)}$ and model $M_r^{(k)}$. Finally, we compute the net benefit, $\mathrm{NB}(d, \boldsymbol{\theta}_r^{(k)}, M_r^{(k)})$ for each d. Repeating this $k = 1, \ldots, K$ times gives us, for each d, a vector of K net benefits, and K corresponding datasets $\tilde{\mathbf{x}}^{(1)}, \ldots, \tilde{\mathbf{x}}^{(K)}$.

We express (for each d) the observed model-averaged net benefit $\mathrm{NB}(d, \boldsymbol{\theta}_r^{(k)}, M_r^{(k)})$ as a sum of the posterior expectation of the net benefit given dataset $\tilde{\mathbf{x}}^{(k)}$ and a mean-zero error term,

$$\mathrm{NB}(d, \boldsymbol{\theta}_r^{(k)}, M_r^{(k)}) = \mathbb{E}_{\boldsymbol{\theta}_r, \mathcal{M}|\mathbf{x}, \tilde{\mathbf{x}}^{(k)}}\{\mathrm{NB}(d, \boldsymbol{\theta}_r, M_r)\} + \varepsilon^{(k)}. \tag{15}$$

We can think of the expectation $\mathbb{E}_{\boldsymbol{\theta}_r, \mathcal{M}|\mathbf{x}, \tilde{\mathbf{x}}^{(k)}}\{\mathrm{NB}(d, \boldsymbol{\theta}_r, M_r)\}$ as an unknown function of $\tilde{\mathbf{x}}^{(k)}$, which we denote $g(d, \tilde{\mathbf{x}}^{(k)})$. Substituting this into Equation (15) gives

$$\mathrm{NB}(d, \boldsymbol{\theta}_r^{(k)}, M_r^{(k)}) = g(d, \tilde{\mathbf{x}}^{(k)}) + \varepsilon^{(k)}. \tag{16}$$

This means that the posterior model-averaged net benefit can be expressed in terms of a single function g and error term ε, independent of the number of models m considered in the analysis. We write the the function g in terms of a low-dimensional summary statistic of the survival data $T(\tilde{\mathbf{x}}^{(k)})$,

$$\mathrm{NB}(d, \boldsymbol{\theta}_r^{(k)}, M_r^{(k)}) = g\{d, T(\tilde{\mathbf{x}}^{(k)})\} + \varepsilon^{(k)}. \tag{17}$$

We then estimate the posterior model-averaged net benefit as before, by fitting a GAM model to each decision option d and extracting the regression model fitted values $\hat{g}_d^{(k)}$. The model-averaged EVSI is then given by Equation (9).

The GAM regression-based scheme for computing model-averaged EVSI is given in Box 2.

Box 2: Generalized Additive Model (GAM) Regression-Based Scheme for Computing Model-Averaged EVSI

for $k = 1, \ldots, K$ outer loops **do**

- Sample a model $M_r^{(k)}$ given current data $\mathbf{x}$ with probability $P(M_r|\mathbf{x})$
- Sample $\boldsymbol{\theta}_r^{(k)}$ from the distribution of the parameters of the sampled model, $p(\boldsymbol{\theta}_r|\mathbf{x}, M_r^{(k)})$
- Evaluate net benefit $\mathrm{NB}(d, \boldsymbol{\theta}_r^{(k)}, M_r^{(k)})$
- Generate a new data sample $\tilde{\mathbf{x}}^{(k)}$ from the distribution of the data $p(\tilde{\mathbf{x}}|\boldsymbol{\theta}_r^{(k)}, M_r^{(k)})$
- Calculate a summary statistic $T(\tilde{\mathbf{x}}^{(k)})$

end

Regress the net benefits $\mathrm{NB}(d, \boldsymbol{\theta}_r^{(k)}, M_r^{(k)})$ on $T(\tilde{\mathbf{x}}^{(k)})$ for each decision option d using GAM
Extract the GAM fitted values $\hat{g}_d^{(k)}$ for each d
Compute EVSI via Equation (9)

Appendix E - Truncated likelihood functions for the Weibull, Gamma, Lognormal and Log-logistic survival models

Note that we use the same parameterisations of the Weibull, Gamma, Lognormal and Log-logistic distributions as in the R package `flexsurv`.[2]

Truncated likelihood function for the Weibull distribution

In order to compute EVSI via the nested Monte Carlo scheme described by Equation (5) we need to define the Weibull truncated likelihood functions for the generated data: $p(\tilde{\mathbf{x}}_1^{(k)}|\theta_{k1}, \theta_{\lambda 1})$ for new treatment and $p(\tilde{\mathbf{x}}_2^{(k)}|\theta_{k2}, \theta_{\lambda 2})$ for standard care.

The Weibull hazard function for the new treatment arm given log-shape θ_{k1}, log-scale $\theta_{\lambda 1}$ and survival time x is

$$h(x,\theta) = \frac{e^{\theta_{k1}}}{e^{\theta_{\lambda 1}}} \left(\frac{x}{e^{\theta_{\lambda 1}}} \right)^{e^{\theta_{k1}}-1}. \tag{18}$$

The survivor function is

$$S(x,\theta) = e^{-\left(x/e^{\theta_{\lambda 1}}\right)^{e^{\theta_{k1}}}}, \tag{19}$$

and the left-truncated likelihood function is therefore

$$\text{Left-truncated likelihood } p(\tilde{\mathbf{x}}|\theta_{k1},\theta_{\lambda 1}) = \prod_{i=1}^{n_2} \left[\frac{\left\{ \frac{e^{\theta_{k1}}}{e^{\theta_{\lambda 1}}} \left(\frac{\tilde{x}_i}{e^{\theta_{\lambda 1}}} \right)^{e^{\theta_{k1}}-1} \right\}^{\tilde{\delta}_i} e^{-\left(\tilde{x}_i/e^{\theta_{\lambda 1}}\right)^{e^{\theta_{k1}}}}}{e^{-\left(t_1/e^{\theta_{\lambda 1}}\right)^{e^{\theta_{k1}}}}} \right]. \tag{20}$$

where $\tilde{x}_i$ and $\tilde{\delta}_i$ are the survival time and censoring indicator for patient i, where censoring is at the proposed new follow-up time of t_2. The expressions above are similarly defined for standard care ($d = 2$) with $\theta_{k2}, \theta_{\lambda 2}$ replacing $\theta_{k1}, \theta_{\lambda 1}$, and $\tilde{\mathbf{x}}_2$ replacing $\tilde{\mathbf{x}}_1$.

Let i index the $n_1 = N$ study participants at risk at time zero, where the censoring indicator $\delta_i = 1$ when x_i is an observed event, $\delta_i = 0$ when x_i is a censored observation, and where $\boldsymbol{\theta}$ are the parameters of the survival distribution. The observed dataset at time point t_1 consists of the n_1 survival times and censoring indicators, $\mathbf{x} = \{x_1, \ldots, x_{n_1}, \delta_1, \ldots, \delta_{n_1}\}$. Denote the data collected between time points t_1 and t_2 as $\tilde{\mathbf{x}} = \{\tilde{x}_1, \ldots, \tilde{x}_{n_2}, \tilde{\delta}_1, \ldots, \tilde{\delta}_{n_2}\}$, where n_2 is the number of study participants at risk at t_1. Events occurring between t_1 and t_2 are conditional on not having occurred before t_1.

Truncated likelihood function for the Gamma distribution

The Gamma density function given log-shape θ_α, log-rate θ_β and survival time x is

$$f(x,\theta) = \frac{(e^{\theta_\beta})^{e^{\theta_\alpha}}}{\Gamma(e^{\theta_\alpha})} x^{e^{\theta_\alpha}-1} e^{-xe^{\theta_\beta}}.$$

The survivor function is

$$S(x,\theta) = 1 - \gamma(e^{\theta_\alpha}, x),$$

where $\gamma(e^{\theta_\alpha}, x)$ is the lower incomplete gamma function, given by

$$\gamma(e^{\theta_\alpha}, x) = \frac{1}{\Gamma(e^{\theta_\alpha})} \int_0^x u^{e^{\theta_\alpha}-1} e^{-u} \mathrm{d}u.$$

We can define the left-truncated likelihood function for the Gamma distribution in terms of the density function and survivor function,

$$\begin{aligned} \text{Left-truncated likelihood } p(\tilde{\mathbf{x}}|\theta_\alpha,\theta_\beta) &= \prod_{i=1}^{n_2} \left[\frac{f(\tilde{x}_i,\theta_\alpha,\theta_\beta)^{\tilde{\delta}_i} S(\tilde{x}_i,\theta_\alpha,\theta_\beta)^{1-\tilde{\delta}_i}}{S(t_1,\theta_\alpha,\theta_\beta)} \right] \\ &= \prod_{i=1}^{n_2} \left[\frac{\left\{ \frac{(e^{\theta_\beta})^{e^{\theta_\alpha}}}{\Gamma(e^{\theta_\alpha})} \tilde{x}_i^{e^{\theta_\alpha}-1} e^{-\tilde{x}_i e^{\theta_\beta}} \right\}^{\tilde{\delta}_i} \left\{ 1 - \gamma(e^{\theta_\alpha}, \tilde{x}_i) \right\}^{1-\tilde{\delta}_i}}{1 - \gamma(e^{\theta_\alpha}, t_1)} \right]. \end{aligned}$$

Truncated likelihood function for the Lognormal distribution

The Lognormal density function given mean θ_μ and log-standard deviation θ_σ of the logarithm, and survival time x is

$$f(x,\theta) = \frac{1}{xe^{\theta_\sigma}\sqrt{2\pi}} e^{-\left(\frac{(\log x - \theta_\mu)^2}{2(e^{\theta_\sigma})^2}\right)}.$$

The survivor function is

$$S(x,\theta) = 1 - \Phi\left(\frac{\log x - \theta_\mu}{e^{\theta_\sigma}}\right),$$

where Φ is the cumulative distribution function of the standard normal distribution $\mathcal{N}(0,1)$.

The hazard function is given by

$$\begin{aligned} h(x,\theta) &= \frac{f(x,\theta)}{S(x,\theta)} \\ &= \frac{\frac{1}{xe^{\theta_\sigma}\sqrt{2\pi}} e^{-\left(\frac{(\log x - \theta_\mu)^2}{2(e^{\theta_\sigma})^2}\right)}}{1 - \Phi\left(\frac{\log x - \theta_\mu}{e^{\theta_\sigma}}\right)} \end{aligned}$$

and the left-truncated likelihood function is

$$\text{Left-truncated likelihood } p(\tilde{\mathbf{x}}|\theta_\mu,\theta_\sigma) = \prod_{i=1}^{n_2} \left[\frac{\left\{ \frac{\frac{1}{\tilde{x}_i e^{\theta_\sigma}\sqrt{2\pi}} e^{-\left(\frac{(\log \tilde{x}_i - \theta_\mu)^2}{2(e^{\theta_\sigma})^2}\right)}}{1-\Phi\left(\frac{\log \tilde{x}_i - \theta_\mu}{e^{\theta_\sigma}}\right)} \right\}^{\tilde{\delta}_i} \left\{1 - \Phi\left(\frac{\log \tilde{x}_i - \theta_\mu}{e^{\theta_\sigma}}\right)\right\}}{1 - \Phi\left(\frac{\log t_1 - \theta_\mu}{e^{\theta_\sigma}}\right)} \right].$$

Truncated likelihood function for the Log-logistic distribution

The Log-logistic hazard function given log-shape θ_s, log-scale θ_η and survival time x is

$$h(x,\theta) = \frac{\frac{e^{\theta_s}}{e^{\theta_\eta}}\left(\frac{x}{e^{\theta}_{\eta}}\right)^{e^{\theta_s}-1}}{1 + \left(\frac{x}{e^{\theta_\eta}}\right)^{e^{\theta_s}}},$$

the survivor function is

$$S(x,\theta) = \frac{1}{1 + \left(\frac{x}{e^{\theta_\eta}}\right)^{e^{\theta_s}}},$$

and the left-truncated likelihood function is

$$\text{Left-truncated likelihood } p(\tilde{\mathbf{x}}|\theta_s, \theta_\eta) = \prod_{i=1}^{n_2} \left[\frac{ \left\{ \frac{ \frac{e^{\theta_s}}{e^{\theta_\eta}} \left(\frac{\tilde{x}_i}{e^{\theta}_{\eta}} \right)^{e^{\theta_s}-1} }{ 1+\left(\frac{\tilde{x}_i}{e^{\theta_\eta}} \right)^{e^{\theta_s}} } \right\}^{\tilde{\delta}_i} \frac{1}{1+\left(\frac{\tilde{x}_i}{e^{\theta_\eta}} \right)^{e^{\theta_s}}} }{ \frac{1}{1+\left(\frac{t_1}{e^{\theta_\eta}} \right)^{e^{\theta_s}}} } \right].$$

Method for sampling from a truncated distribution

We can sample values from a truncated survival distribution that lie in the interval (t_1, ∞) as follows. We denote the cumulative density function evaluated at time t with parameters $\boldsymbol{\theta}$ as $F(t, \boldsymbol{\theta})$. We first compute the value of the cumulative density function at t_1, $p = F(t_1, \boldsymbol{\theta})$, (i.e. the probability that a survival time will exceed t_1). We then sample n values from a uniform distribution on the interval $[p, 1]$, and plug these into the corresponding *inverse* cumulative density function $F^{-1}(\cdot, \boldsymbol{\theta})$. This results in n survival times greater than t_1 that follow the required truncated survival distribution.

Appendix F - The impact of increasing follow-up durations on the standard errors of the MCMC and GAM estimators

Increasing follow-up durations affect the standard errors of the nested Monte Carlo and GAM estimators in different ways. A longer duration of additional follow-up time will result in a greater effective sample size (ESS) of the generated data $\tilde{\mathbf{x}}$, as the number of observed events e and time at risk y will be greater. When ESS $\rightarrow 0$, the posterior expectation $\mathbb{E}_{\boldsymbol{\theta}|\mathbf{x},\tilde{\mathbf{x}}^{(k)}}$ will be similar to the prior expectation $\mathbb{E}_{\boldsymbol{\theta}}$ for all k, and the variance of the posterior mean will therefore tend to 0. When ESS $\rightarrow \infty$, the posterior expectation $\mathbb{E}_{\boldsymbol{\theta}|\mathbf{x},\tilde{\mathbf{x}}^{(k)}}$ will be similar to the prior parameter sample $\boldsymbol{\theta}^{(k)}$ that was used to generate the data $\tilde{\mathbf{x}}^{(k)}$ for all k, and the variance of the posterior mean will therefore converge to the variance of $\boldsymbol{\theta}$. Thus, as the variance of the posterior mean increases with increasing values for the additional follow-up time, the standard error of the nested Monte Carlo estimator is expected to increase as well. The relation between the posterior and prior variance as a function of sample size is further explained in a paper by Jalal & Alarid-Escudero (2018).[3]

The ESS affects the standard error of the GAM estimator differently. We recall that the GAM approach relies on expressing the posterior expected net benefit as a function of the generated data $\tilde{\mathbf{x}}$. When ESS $\rightarrow \infty$, the variance of the error term $\varepsilon^{(k)}$ in the expression $\text{NB}(d, \boldsymbol{\theta}^{(k)}) = \mathbb{E}_{\boldsymbol{\theta}|\mathbf{x},\tilde{\mathbf{x}}^{(k)}}\{\text{NB}(d, \boldsymbol{\theta})\} + \varepsilon^{(k)}$ will tend to 0, since the posterior expectation $\mathbb{E}_{\boldsymbol{\theta}|\mathbf{x},\tilde{\mathbf{x}}^{(k)}}$ will be similar to the prior parameter sample $\boldsymbol{\theta}^{(k)}$ that was used to generate the data $\tilde{\mathbf{x}}^{(k)}$ for all k. The smaller the variance of the error term $\varepsilon^{(k)}$, the greater the precision with which the GAM regression coefficients can be estimated, and the smaller the variance of the regression fitted values. Increasing the length of additional follow-up time increases the precision with which the GAM regression coefficients are estimated, and consequently reduces the standard error of the GAM estimator.

Appendix G - Maximum likelihood estimates for the model parameters

Table G1: Bivariate Normal distribution hyperparameters for the Weibull model parameters given data collected up to $t_1 = 12$ months

Parameter		Mean, $\boldsymbol{\mu}$	Covariance matrix, $\boldsymbol{\Sigma}$
Case study 1: Increasing hazard dataset			
Log shape for new treatment<br>Log scale for new treatment	$\begin{pmatrix}\theta_{k1}\\ \theta_{\lambda 1}\end{pmatrix}$	$\begin{pmatrix}0.275\\ 4.014\end{pmatrix}$	$\begin{pmatrix}0.039 & -0.060\\ -0.060 & 0.117\end{pmatrix}$
Log shape for standard care<br>Log scale for standard care	$\begin{pmatrix}\theta_{k2}\\ \theta_{\lambda 2}\end{pmatrix}$	$\begin{pmatrix}0.257\\ 3.863\end{pmatrix}$	$\begin{pmatrix}0.031 & -0.044\\ -0.044 & 0.081\end{pmatrix}$
Case study 2: Decreasing hazard dataset			
Log shape for new treatment<br>Log scale for new treatment	$\begin{pmatrix}\theta_{k1}\\ \theta_{\lambda 1}\end{pmatrix}$	$\begin{pmatrix}-0.392\\ 4.472\end{pmatrix}$	$\begin{pmatrix}0.020 & -0.043\\ -0.043 & 0.136\end{pmatrix}$
Log shape for standard care<br>Log scale for standard care	$\begin{pmatrix}\theta_{k2}\\ \theta_{\lambda 2}\end{pmatrix}$	$\begin{pmatrix}-0.412\\ 4.331\end{pmatrix}$	$\begin{pmatrix}0.018 & -0.036\\ -0.036 & 0.115\end{pmatrix}$

Table G2: Bivariate Normal distribution hyperparameters for the Gamma model parameters given data collected up to $t_1 = 12$ months

Parameter		Mean, $\boldsymbol{\mu}$	Covariance matrix, $\boldsymbol{\Sigma}$
Case study 1: Increasing hazard dataset			
Log shape for new treatment<br>Log rate for new treatment	$\begin{pmatrix}\theta_{\alpha 1}\\ \theta_{\beta 1}\end{pmatrix}$	$\begin{pmatrix}0.310\\ -3.752\end{pmatrix}$	$\begin{pmatrix}0.051 & 0.114\\ 0.114 & 0.279\end{pmatrix}$
Log shape for standard care<br>Log rate for standard care	$\begin{pmatrix}\theta_{\alpha 2}\\ \theta_{\beta 2}\end{pmatrix}$	$\begin{pmatrix}0.291\\ -3.612\end{pmatrix}$	$\begin{pmatrix}0.042 & 0.088\\ 0.088 & 0.208\end{pmatrix}$
Case study 2: Decreasing hazard dataset			
Log shape for new treatment<br>Log rate for new treatment	$\begin{pmatrix}\theta_{\alpha 1}\\ \theta_{\beta 1}\end{pmatrix}$	$\begin{pmatrix}-0.434\\ -4.861\end{pmatrix}$	$\begin{pmatrix}0.024 & 0.067\\ 0.067 & 0.230\end{pmatrix}$
Log shape for standard care<br>Log rate for standard care	$\begin{pmatrix}\theta_{\alpha 2}\\ \theta_{\beta 2}\end{pmatrix}$	$\begin{pmatrix}-0.458\\ -4.752\end{pmatrix}$	$\begin{pmatrix}0.022 & 0.059\\ 0.059 & 0.198\end{pmatrix}$

Table G3: Bivariate Normal distribution hyperparameters for the Lognormal model parameters given data collected up to $t_1 = 12$ months

Parameter		Mean, $\boldsymbol{\mu}$	Covariance matrix, $\boldsymbol{\Sigma}$
Case study 1: Increasing hazard dataset			
Meanlog for new treatment<br>Log sdlog for new treatment	$\begin{pmatrix}\theta_{\mu 1}\\ \theta_{\sigma 1}\end{pmatrix}$	$\begin{pmatrix}4.366\\ 0.488\end{pmatrix}$	$\begin{pmatrix}0.164 & 0.062\\ 0.062 & 0.029\end{pmatrix}$
Meanlog for standard care<br>Log sdlog for standard care	$\begin{pmatrix}\theta_{\mu 2}\\ \theta_{\sigma 2}\end{pmatrix}$	$\begin{pmatrix}4.133\\ 0.477\end{pmatrix}$	$\begin{pmatrix}0.113 & 0.044\\ 0.044 & 0.023\end{pmatrix}$
Case study 2: Decreasing hazard dataset			
Meanlog for new treatment<br>Log sdlog for new treatment	$\begin{pmatrix}\theta_{\mu 1}\\ \theta_{\sigma 1}\end{pmatrix}$	$\begin{pmatrix}4.622\\ 1.047\end{pmatrix}$	$\begin{pmatrix}0.185 & 0.041\\ 0.041 & 0.015\end{pmatrix}$
Meanlog for standard care<br>Log sdlog for standard care	$\begin{pmatrix}\theta_{\mu 2}\\ \theta_{\sigma 2}\end{pmatrix}$	$\begin{pmatrix}4.395\\ 1.045\end{pmatrix}$	$\begin{pmatrix}0.157 & 0.034\\ 0.034 & 0.013\end{pmatrix}$

Table G4: Bivariate Normal distribution hyperparameters for the Log-logistic model parameters given data collected up to $t_1 = 12$ months

Parameter		Mean, $\boldsymbol{\mu}$	Covariance matrix, $\boldsymbol{\Sigma}$
Case study 1: Increasing hazard dataset			
Log shape for new treatment<br>Log scale for new treatment	$\begin{pmatrix}\theta_{s 1}\\ \theta_{\eta 1}\end{pmatrix}$	$\begin{pmatrix}0.308\\ 3.915\end{pmatrix}$	$\begin{pmatrix}0.038 & -0.056\\ -0.056 & 0.107\end{pmatrix}$
Log shape for standard care<br>Log scale for standard care	$\begin{pmatrix}\theta_{s 2}\\ \theta_{\eta 2}\end{pmatrix}$	$\begin{pmatrix}0.297\\ 3.748\end{pmatrix}$	$\begin{pmatrix}0.030 & -0.040\\ -0.040 & 0.074\end{pmatrix}$
Case study 2: Decreasing hazard dataset			
Log shape for new treatment<br>Log scale for new treatment	$\begin{pmatrix}\theta_{s 1}\\ \theta_{\eta 1}\end{pmatrix}$	$\begin{pmatrix}-0.331\\ 4.173\end{pmatrix}$	$\begin{pmatrix}0.019 & -0.037\\ -0.037 & 0.123\end{pmatrix}$
Log shape for standard care<br>Log scale for standard care	$\begin{pmatrix}\theta_{s 2}\\ \theta_{\eta 2}\end{pmatrix}$	$\begin{pmatrix}-0.343\\ 4.002\end{pmatrix}$	$\begin{pmatrix}0.017 & -0.030\\ -0.030 & 0.104\end{pmatrix}$

References

1. Strong M, Oakley JE, Brennan A, Breeze P. Estimating the Expected Value of Sample Information Using the Probabilistic Sensitivity Analysis Sample: A Fast, Nonparametric Regression-Based Method. Medical Decision Making. 2015 Jul;35(5):570–583.
2. Jackson C. Flexsurv: A Platform for Parametric Survival Modeling in R. Journal of Statistical Software. 2016 May;70(1):1–33.
3. Jalal H, Alarid-Escudero F. A Gaussian Approximation Approach for Value of Information Analysis. Medical Decision Making. 2018 Feb;38(2):174–188.
